# Improving the Quality and Safety of Fish Products with Edible Coatings Incorporating Piscicolin CM22 from a Psychrotolerant *Carnobacterium maltaromaticum* Strain

**DOI:** 10.3390/foods13193165

**Published:** 2024-10-04

**Authors:** Elías González-Gragera, José David García-López, Abdelkader Boutine, María Luisa García-Marín, Juristo Fonollá, Lidia Gil-Martínez, Inmaculada Fernández, Manuel Martínez-Bueno, Alberto Baños

**Affiliations:** 1 Department of Microbiology, University of Granada, Avda. Fuentenueva, s/n, 18071 Granada, Spain; elias.es@hotmail.com (E.G.-G.); mluisagmarin@gmail.com (M.L.G.-M.); mmartine@ugr.es (M.M.-B.); 2 Department of Microbiology, DMC Research Center, Camino de Jayena s/n, 18620 Granada, Spain; jdgarcia@correo.ugr.es (J.D.G.-L.); aboutine@dmcrc.com (A.B.); lidiagm@domca.com (L.G.-M.); ifernandez@domca.com (I.F.); 3 Department of Nutrition and Food Technology, University of Granada, 18071 Granada, Spain; 4 Institute of Biotechnology, University of Granada, 18071 Granada, Spain

**Keywords:** piscicolin CM22, *Listeria*, raw fish, smoked salmon, edible coating, biopreservation

## Abstract

The consumption of raw or smoked fish entails significant microbiological risks, including contamination by pathogens such as *Listeria monocytogenes*, which can cause severe foodborne illnesses. This study explores the preservative use of piscicolin CM22, a novel bacteriocin derived from the psychrotolerant strain *Carnobacterium maltaromaticum* CM22, in two types of edible coatings (EC): chitosan-based and fish gelatin-based. An initial in vitro characterization of the technological and antimicrobial properties of these ECs with and without bacteriocin was conducted. The efficacy of the edible coatings was subsequently evaluated through shelf life and challenge tests against *L. monocytogenes* in raw and smoked fish products. The results demonstrated significant antimicrobial activity, with the chitosan-based coating containing piscicolin CM22 being the most effective in reducing microbial counts and maintaining pH and color stability. Furthermore, in the challenge test studies, both ECs effectively controlled *L. monocytogenes*, showing significant reductions in bacterial counts compared to the controls in fresh tuna, salmon, and smoked salmon. The ECs containing piscicolin CM22 reduced *Listeria* counts by up to 4 log CFU/g in raw and smoked fish samples, with effective control in smoked salmon for up to 15 days at refrigeration temperature. While further research is required to fully assess their preservation potential, these findings strongly indicate that piscicolin CM22-functionalized edible coatings hold significant potential for improving the quality and safety of fish products.

## 1. Introduction

The contamination of fish and seafood by pathogenic microorganisms is a significant public health concern. Numerous studies have reported a high prevalence of *Listeria monocytogenes* in both raw and processed fish and seafood, conclusively identifying these foods as potential carriers of this pathogen [1]. Common routes for contamination of fish with *Listeria* include its spread from intestinal contents to other tissues, as well as cross-contamination through contact with various surfaces and processing equipment, such as conveyor belts, trays, and skinning and slicing machines [2]. The current strategies for controlling *L. monocytogenes* in fish and seafood include chemical treatments such as preservatives, chlorinated products, ozone, or electrolyzed oxidizing water, among others [3,4]. In addition, physical treatments such as steam application or pulsed light exposure have also been evaluated [5,6]. However, very few technologies achieve total *Listeria* elimination, highlighting the need for continued research into new preservation methods that ensure its control.

Among the emerging methods for *Listeria* control are biopreservation strategies, which employ microorganisms and/or their metabolites, such as antimicrobial peptides or bacteriocins [7]. In this regard, the use of bacteriocins from lactic acid bacteria (LAB) could represent an effective and cost-efficient alternative to help reduce the incidence of this pathogen in fish and seafood products [8]. In fact, some authors have described the potential of several bacteriocins from LAB, such as enterocin AS-48, nisin, or piscicolin, among others [9,10,11,12], for controlling *Listeria* in fish products.

Piscicolin CM22, a new cationic bacteriocin similar to piscicolin 126, is produced by the psychrotolerant strain *Carnobacterium maltaromaticum* CM-22, which was previously isolated by our team from salmon (*Salmo salar*). This bacteriocin exhibits strong bactericidal activity against several Gram-positive bacteria, including *L. monocytogenes* [13,14,15]. As with other piscicolins, CM22 is anticipated to effectively control this pathogen in food products. However, given that contact surfaces are the primary source of microbiological contamination in the fish industry [16,17], it is crucial to optimize the application methods of these bacteriocins for surface treatments, such as direct spraying or its incorporation into edible coatings.

Edible coatings (ECs) are food-grade suspensions that may be delivered by spraying, spreading, or dipping, which upon drying form a thin layer on the food surface. They have been proven to be able to extend the shelf life and improve the safety of food in different ways. Furthermore, ECs are used to improve the appearance and mechanical properties of food products, reducing water loss [18,19]. While the primary function of edible coatings is to serve as a physical barrier to protect food from gases and dehydration, in recent years, ECs have also begun to be used as carriers for functional ingredients, such as preservatives [20].

Considerable research has been conducted, providing insights into the potential utilization of several food ingredients such as chitosan and plant extracts, for incorporation into edible coatings to enhance their antimicrobial effectiveness [21,22,23]. Bacteriocins such as piscicolins are also promising candidates for edible coatings (ECs) due to their natural preservative qualities. Additionally, they are recognized as safe (GRAS) and are typically produced by LAB, making them suitable for food applications without compromising consumer safety. Furthermore, the incorporation of bacteriocins into ECs would enhance the antimicrobial barrier properties of these coatings and reduce the reliance on chemical preservatives. This approach addresses consumer demand for cleaner labels and more natural food products. In summary, the integration of bacteriocins into ECs represents an advanced biopreservation strategy that leverages the natural antimicrobial properties of these peptides to improve food safety and quality [24].

This study aimed to evaluate the effectiveness of incorporating piscicolin CM22 into two types of edible coatings (chitosan-based and fish gelatin-based) for the preservation and control of *Listeria* species in fish products during refrigerated storage. This study innovatively integrates piscicolin CM22, a scarcely studied bacteriocin, into these edible coatings, offering an effective and natural solution to enhance the safety and extend the shelf life of raw and smoked fish products.

## 2. Materials and Methods

### 2.1. Bacterial Strains and Culture Conditions

*Carnobacteirum maltaromaticum* CM22 [13], from our collection, was used as the piscicolin CM22 producer. *L. monocytogenes* CECT 4032 supplied by the Spanish Type Culture Collection (CECT) was used as the standard indicator strain for bacteriocin activity assays. *C. maltaromaticum* CM22 was grown in BHI Brain Heart Infusion broth (Sharlau, Barcelona, Spain) at 28 °C for 24 h. For fish inoculation, *L. monocytogenes* DSM 112143 isolated from smoked organic salmon were obtained from the German Collection of Microorganisms and Cell Cultures (DSMZ), *Listeria seeligeri* CECT 5342 isolates from smoked fish *L. monocytogenes* DMC 05 and *Listeria innocua* DMC 04 isolated from raw salmon from our collection were used to obtain more detailed information about the anti-Listeria activity of piscicolin CM22. *Listeria* strains were grown overnight on brain heart infusion (BHI) (Sharlau, Barcelona, Spain) at 37 °C, washed in a sterile saline solution, and then inoculated in fillet tissue samples at the selected concentration. All bacterial cultures were maintained at 4 °C on BHI agar (BHA) slants.

### 2.2. Preparation of Piscicolin CM22

Piscicolin CM22 was obtained by culturing the strain *C. maltaromaticum* CM22 in a food grade substrate including saccharose (1%), yeast extract (0.3%), NaCl (1%), lactose (1%), and 1 mM MgSO_4_. Cation exchange chromatography on carboxymethyl Sephadex CM-25 (Sigma Aldrich, St. Louis, MO, USA) was used to recover piscicolin from cultures. Bacteriocin activity against the indicator strain *L. innocua* was tested by eluting and using the agar well diffusion method [25]. NaCl was eliminated before the use of the eluted fractions: they were dialyzed at 4 °C against distilled water through a 2000-Da cut-off membrane, and then sterilized by filtration (0.22 μm, Milli-pore, Belford, MA, USA). The bacteriocin was quantified and adjusted to achieve a final concentration of 10,240 AU/mL according to the method described by González-Gragera et al. (2024) [13]. 

### 2.3. Enzymatic and Physicochemical Stability of Piscicolin CM22

To investigate the technological applications of piscicolin CM22 in food preservation and its potential incorporation into edible coatings (ECs), its enzymatic, thermal, and pH stability were studied. Enzymatic stability was assessed using the methodology described by Zhao et al. (2020) [26]. For this purpose, papain (≥10 units/mg, Sigma, St. Louis, MO, USA), trypsin (2000 units/mg, Sigma, St. Louis, MO, USA), and proteinase K (30 units/mg, Sigma, St. Louis, MO, USA) were used. The bacteriocin was mixed with each of the enzymes at a concentration of 0.5 mg/mL at 37 °C during 2 h. Afterward, the enzymes were inactivated by heat at 80 °C for 5 min. In addition, pH and thermal stability were tested using the methodology proposed by Ahn et al. (2017) [27]. To test thermal stability, the pH of purified bacteriocin was adjusted to 6.5 using HCl 4N or NaOH, and then three aliquots were heated to 60, 80, and 100 °C for 30 min each, and one of them was autoclaved (121 °C, 15 min). To test pH stability, the pH of bacteriocin was adjusted to values of 2, 3, 5, 7, 9, and 11 with HCl 4N or NaOH and incubated for 2 h at 30 °C. Finally, before assessing the antimicrobial activity of each sample against *L. monocytogenes*, the piscicolin solution was neutralized to pH 6.5.

### 2.4. Edible Coatings (ECs)

Two edible coatings were prepared for the incorporation of piscicolin CM22.

Fish gelatine-based coating (GC). This coating was prepared using cold-water-soluble fish gelatin (Norland Fish Products, Cranbury, NJ, USA). The film-forming solution was formulated to contain 4.5% gelatin, 1.5% glycerol as a plasticizer, and 1% sodium alginate (Sigma, St. Louis, MO, USA). First, 10 g of sodium alginate was slowly incorporated, with stirring (200 rpm), into a beaker containing 500 mL of warm deionized water. Once the sodium alginate was fully dissolved in the deionized water, 15 g of glycerol were added, continuing to stir the solution (200 rpm) for 10 min. After cooling the solution to 4 °C, fish gelatin and piscicolin CM22, previously dissolved in 430 mL of deionized water, were added to reach a concentration of 1000 AU/mL, followed by stirring (450 rpm for 10 min). In addition, a coating without bacteriocin was prepared as a control (GC). Both the control and the edible coatings containing piscicolin CM22 (GC-CM22) were stored under refrigeration until use.

Chitosan-based coating (CC). A chitosan coating was prepared by dissolving 18 g of chitosan with a deacetylation degree of 90% (Sigma, St. Louis, MO, USA) in 1 Kg of a sodium diacetate (2% *w*/*w*) (Brenntag, Reading, PA, USA) to obtain the acidic conditions (pH = 5.1), which was needed to dissolve the biopolymer. It was added by rapid stirring (450 rpm) to obtain an opalescent solution. The solution was stirred using a magnetic stirrer at a rapid stirring speed (450 rpm) to achieve an opalescent solution. The sample was allowed to stand overnight to achieve the complete solution of chitosan and then filtered through a sterilized glass filter (Whatman, UK). Finally, the sample was added to piscicolin CM22 until reaching a concentration of 1000 AU/mL (CC-CM22). Similarly, a control coating without bacteriocin was formulated (CC). All coatings were stored refrigerated until needed.

### 2.5. Physicochemical Characterization of Coatings

An initial physicochemical characterization was performed on each edible coating, involving the assessment of the following parameters:

Thickness of the films (TFs). The thickness of the films was measured using a micro caliper at six different points of the same sample. Results were expressed as the average ± SD.

Films solubility (FS). For the determination of the solubility in water of the film, first, a square film of 2.5 cm^2^ was dried at 80 °C for 24 h and then weighed to determine the initial dry mass (M_0_). Afterward, the dried films were immersed in 50 mL of distilled water in constant agitation for 12 h at 25 °C. The mixture was centrifuged (4500 rpm, 15 min) and the precipitate was dried at 80 °C for 24 h and weighed to determine the final dry mass (M_f_). Film solubility was determined by Equation (1):

FS % = (M_0_ − M_f_)/M_0_ × 100 (1)

Films water swelling (FWS). The film swelling rate was calculated by the methodology described by Tan et al. 2022 with some modifications [28]. Briefly, the films were dried at 60 °C over 24 h and immersed in 50 mL of distilled water for 24 h. After, the film was removed from the vase, leftover water was dried with filter paper, and the films were weighed (M_w_). Then, the films were dried at 60 °C until constant weight (Mf). The water swelling rate was determined according to Equation (2):

FWS% = (M_w_ − M_f_)/M_w_ × 100 (2)

Films water vapor permeability (WVP). Water vapor permeability was measured according to the methodology described by Ma et al. 2015 with some modifications [29]. Briefly, 2.5 g of anhydrous CaCl_2_ was added into a 50 mm × 30 mm beaker and then covered with the films. The samples were kept in a chamber at 25 °C and 75% of relative humidity. The mass of the bottle was recorded daily for 3 days until constant weight. WVP (mg cm^−2^ h^−1^) was determined using the following Equation (3):

WVP = Δm/(A × Δ_t_) (3)

where Δm is the sample weight difference (mg), Δ_t_ is the time interval (h), and A is the effective area of the films (cm^2^).

### 2.6. In Vitro Antimicrobial Activity of Edible Coatings (ECs)

To determine the in vitro antimicrobial activity of ECs with and without piscicolin CM22, the target strains *L. monocytogenes* DSM 112143, *L. monocytogenes* CECT 4032, *L. seeligeri* CECT 5342, *L. monocytogenes* DMC 05, and *L. innocua* DMC 04 were used. First, the agar well diffusion method was carried out following the procedure described by Tagg and MacGiven (1979) [30]. Stainless steel cylinders with a diameter of 8 mm and a height of 10 mm were placed on plates containing Mueller Hinton Agar (MHA, Scharlau, Barcelona, Spain) (10 mL). Next, 6 mL of soft BHA (BHA plus 0.8% agar) medium inoculated with approximately 10^7^ CFU of bacteria was poured onto the plates. The cylinders were removed after the overcoating layer solidified, and the holes were individually filled with 100 μL of each edible coating. Finally, the plates were incubated aerobically at 37 °C for 24 or 48 h. After incubation, the clear zones were measured using a microcaliper. Each sample was tested in triplicate.

### 2.7. Food Effectiveness Trials

Raw tuna and salmon were purchased from local retailers (Alhendín, Granada, Spain). For each experiment, a series of fish 3 cm^2^ blocks were prepared by slicing iced flesh with a sterile knife. After that, the four treatments that were used were prepared: fish gelatin-based coating (GC), fish gelatine-based coating with piscicolin CM22 (GC-CM22), chitosan-based coating (CC), and chitosan-based coating with piscicolin CM22 (CC-CM22). In addition, an untreated sample was used as a control. After preparing the treatments, the fish blocks were submerged by dipping for 30 s. Then, the different samples were placed on polystyrene dishes and sealed in a Ziploc bag in modified atmosphere packaging (MAP) (30% CO_2_, 70% N_2_) (Tecnotrip, Barcelona, Spain), and immediately stored at 4 °C and 8 °C. Two independent studies were conducted.

#### 2.7.1. Influence on the Shelf Life of Raw Tuna and Salmon

pH. The determination of pH was carried out during sampling each time. For pH determination, 10 mL of the homogenized sample was used. A pH-meter (Model HI 99163, Hanna, Póvoa de Varzim, Portugal) calibrated with buffer solutions at pH 4.0 and 7.0 was used to determine the pH value of the suspension in triplicates.

Color analyses. For instrumental color determination, a colorimeter (Model CR 410, Konica Minolta, Tokyo, Japan) was employed. The color was measured at three different points on the surface of the fish block. Measurement parameters were color space—CIE L*a*b*, light source—D65, opening diameter—50 to 53 mm, and angle of observation—2°. L* represents the clarity or luminosity of the subject measured—100 corresponds to white and 0 corresponds to black. a* is the green/red color component—a* < 0 is green and a* > 0 red. Finally, b* represents the blue/yellow color component—b* > 0 yellow, b* < 0 blue.

Microbiological counts. The evolution of the microbiology for each treatment was determined in duplicate on days 0, 1, and 3. We followed the same sampling process in the tuna and salmon, mixing 25 g of fish with 225 mL of peptone water in a 400 mL plastic sterile bag (VWR Blender Bag, Barcelona, Spain) and then they were homogenized for 1 min in a Masticator blender (IUL, Barcelona, Spain). Subsequently, each sample was collected in 100 mL plastic containers. Afterward, decimal dilutions were prepared and plated on various media, including Plate Count Agar (PCA) at 30 °C for total aerobic mesophiles, PCA at 4 °C for total psychrophiles, MacConkey agar at 30 °C for enterobacteria, Sabouraud agar with chloramphenicol at 30 °C for yeast, and Rose Bengal Agar at 25 °C for fungi (all media were obtained from Sharlab, Barcelona, Spain).

#### 2.7.2. Challenge Test in Food

Challenge test assays were performed in duplicate according to Baños et al. (2016) [31]. Fish were purchased from local retailers. Fish blocks (25 cm^2^) were prepared in the same manner as previously described. Raw tuna and salmon were inoculated with an adjusted concentration of 5 Log10 CFU/mL to obtain a final 10^3^ CFU/cm^2^ concentration from a pool of *L. monocytogenes* (DSM 112143, DMC 05, CECT 5342, DMC 04, CECT 4032), using a sterile handle Digralsky. Afterwards, different fish fillets were dipped in different treatment solutions for 30 s, including batches with ECs (GC and CC) and batches with ECs with added piscicolin CM22 (GC-CM22 and CC-CM22) at a concentration of 1000 AU/mL. Finally, a distilled water treatment was performed as a control. Additionally, challenge tests were conducted on smoked salmon using a similar procedure. The samples were individually placed on polystyrene dishes, MAP-packed for raw fish, and vacuum-packed for smoked salmon (TECNOTRIP, Barcelona, Spain). The samples were then stored at 4 °C for 3 days for raw tuna and salmon, and 28 days for smoked salmon.

For pathogen monitoring, samples from each treatment were withdrawn in triplicate at selected times to determine viable counts of *Listeria* and mixed (1/10, *w*/*v*) with a dilution medium (0.1% peptone, 0.85% NaCl). Homogenization was performed in a Masticator blender (IUL, Barcelona, Spain) for 1 min followed by serial 10-fold dilution and plating on chromogenic selective agar COMPASS Listeria Agar (Biokar Diagnostics, Allone, France). Results were expressed as Log10 CFU/cm^2^ versus time. When it was not possible to quantify the bacteria below the detection limit (<1 Log10 CFU/cm^2^), an investigation was carried out by pre-enrichment in Fraser broth (Biokar Diagnostics), expressing the results as presence or absence.

### 2.8. Statistical Analyses

The statistics were extracted from the results of two independent experiments. In each one, three samples (food tested) for each treatment (*n* = 6) and sampling time were used. Statistical analysis and figures were performed with GraphPad Prism 8.0 software (GraphPad Software Inc., San Diego, CA, USA). Data on microbiological counts were subjected to ANOVA. Error probability values less than 0.05 were considered not significant.

## 3. Results

### 3.1. In Vitro Trials

Before assessing the efficacy of the ECs enhanced with piscicolin CM22, an in vitro characterization of the bacteriocin’s technological properties was conducted. Initially, the stability of the bacteriocin was evaluated under various conditions of temperature, pH, and enzyme treatment. The results presented in Table 1 show that bacteriocin activity was maintained across a wide pH range of 2 to 10, although it was sensitive to protease enzymes such as pepsin, papain, and trypsin. The antimicrobial activity was not affected by heating at 80 °C for 30 min. However, at higher temperatures, its activity began to decrease, with a complete loss of efficacy observed at 121 °C for 15 min. Bacteriocin activity was optimal at pH 5 and pH 7 (23 ± 0.5), demonstrating high stability. Activity decreased significantly at pH 10 and was completely lost at pH 11, indicating instability under highly alkaline conditions. Finally, the bacteriocin was entirely degraded by trypsin, papain, and proteinase K, confirming its susceptibility to proteolytic enzymes.

Following the assessment of bacteriocin stability under different conditions, we developed two types of edible coatings (ECs) to explore their potential applications in food preservation. These coatings were based on fish gelatin (GC) and chitosan (CC), incorporating piscicolin CM22, resulting in the formulations GC-CM22 and CC-CM22, respectively. After formulating the edible coatings, both with and without the bacteriocin, we conducted a comprehensive physicochemical characterization. The results, presented in Table 2, indicate that the incorporation of piscicolin did not adversely affect the technological properties of the ECs. Specifically, attributes such as film morphology, thickness, solubility, water film swelling, and water vapor permeability remained unaffected.

Subsequently, the antimicrobial activity of both edible coatings (ECs), with and without the incorporation of piscicolin CM22, was assessed. Given the inherent antimicrobial properties of certain EC components, initial in vitro assays were conducted to verify this activity and determine any enhancement in effectiveness resulting from the inclusion of the bacteriocin in the coatings.

The antimicrobial effectiveness of ECs against several *L. monocytogenes* strains were assessed, and the results are summarized in Table 3. The fish gelatin coating (GC) alone exhibited no inhibition, while the GC-CM22 formulation, incorporating piscicolin CM22, showed significant antimicrobial activity with inhibition zones ranging from 19 ± 1 mm to 23 ± 1.3 mm. Although the chitosan coating (CC) alone demonstrated modest inhibition against *Listeria*, with inhibition zones ranging from 10 ± 1 mm to 12 ± 1 mm, the combination of chitosan with piscicolin CM22 (CC-CM22) exhibited significantly higher antimicrobial activity, with inhibition zones extending from 26 ± 1.5 mm to 29 ± 2 mm. These results indicate that while chitosan itself has inherent antimicrobial properties, the addition of piscicolin CM22 markedly enhances its antibacterial effectiveness, suggesting a synergistic interaction between the two components.

### 3.2. Shelf-Life Trials

#### 3.2.1. Influence on Microbial Counts

**Figure 1:**
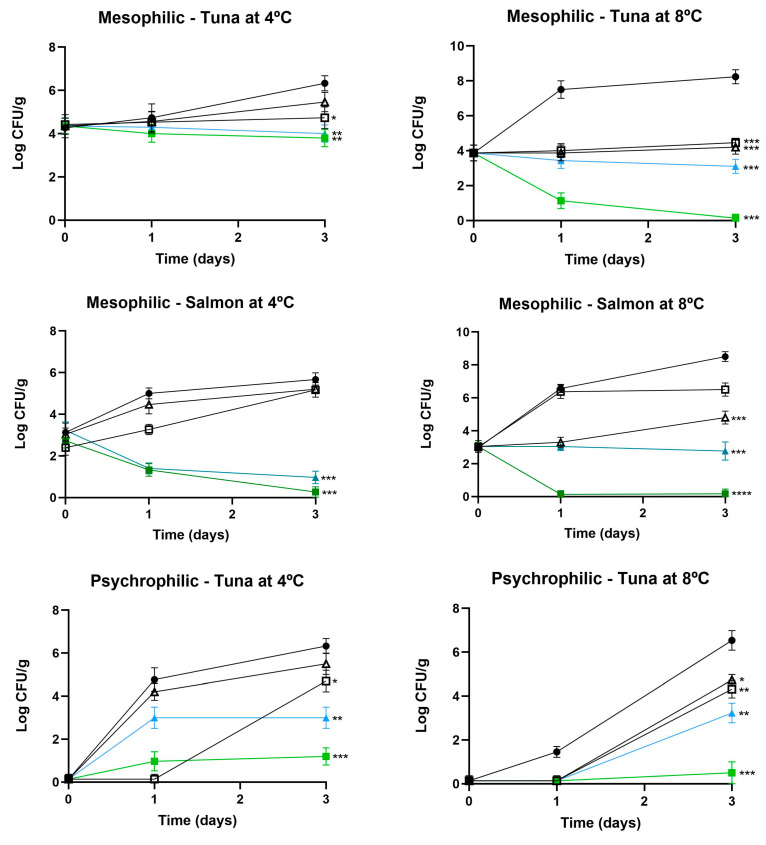

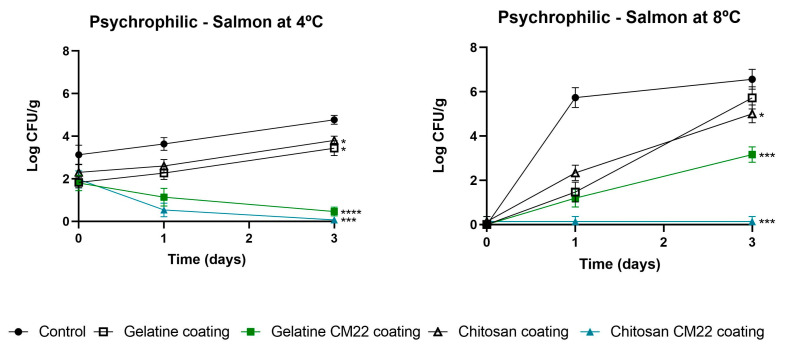
Effect of treatments with different edible coatings (ECs) containing piscicolin CM22 on mesophiles and psychrophiles in raw tuna and salmon stored at 4 °C and 8 °C. Values are the average ± SD (error bars) from independent experiments. Significant differences compared to control: * *p* < 0.05; ** *p* < 0.01; *** *p* < 0.001; **** *p* < 0.0001.

After completing the characterization and in vitro efficacy tests, the effectiveness of the new edible coatings (ECs) in extending the shelf life of raw fish stored at 4 °C and 8 °C was evaluated. Figure 1 illustrates the evolution of microbiological counts for mesophilic and psychrophilic microorganisms in raw tuna and salmon. For mesophilic bacteria, tuna stored at 4 °C showed significant reductions in bacterial counts when treated with gelatin-CM22 and chitosan-CM22 coatings. The chitosan-CM22 coating was the most effective, reducing the counts to approximately 2 log CFU/g on day 3 compared to the control, which reached nearly 6 log CFU/g (*p* < 0.001). Salmon stored at 4 °C exhibited similar trends, with chitosan-CM22 reducing mesophilic counts to about 2 log CFU/g, while the control increased to approximately 5 log CFU/g (*p* < 0.001).

At 8 °C, the effectiveness of the coatings was even more pronounced. Tuna treated with chitosan-CM22 showed a substantial reduction in mesophilic bacteria, with counts remaining around 3 log CFU/g, whereas the control exceeded over 8 log CFU/g (*p* < 0.001). Gelatin-CM22 also significantly inhibited bacterial growth, though not as effectively as chitosan-CM22. In salmon, both coatings demonstrated strong antimicrobial activity, with gelatin-CM22 and chitosan-CM22 reducing mesophilic counts to 3 log CFU/g and 2 log CFU/g, respectively, while the control exceeded 7 log CFU/g (*p* < 0.001).

For psychrophilic microorganisms, tuna stored at 4 °C and 8 °C exhibited significant inhibition when treated with chitosan-CM22, reducing bacterial counts to approximately 1 log CFU/g at both temperatures, compared to controls, which increased to 5 log CFU/g (*p* < 0.001). Gelatin-CM22 also showed effectiveness, reducing counts to about 2 log CFU/g, though not as significantly as chitosan-CM22. Salmon treated with these coatings displayed similar results, with significant reductions in psychrophilic counts at both temperatures, particularly with chitosan-CM22, which maintained levels around 1 log CFU/g, while controls reached 6 log CFU/g (*p* < 0.001).

**Figure 2:**
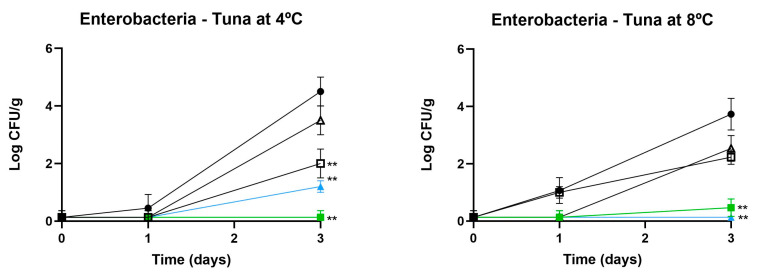

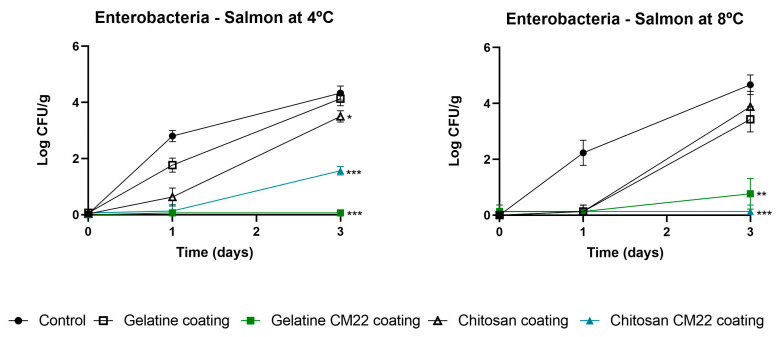
Effect of treatments with different edible coatings (ECs) containing piscicolin CM22 on enterobacteria in raw tuna and salmon stored at 4 °C and 8 °C. Values are the average ± SD (error bars) from independent experiments. Significant differences compared to control: * *p* < 0.05; ** *p* < 0.01; *** *p* < 0.001.

In the case of enterobacteria, the results in Figure 2 show a significant impact of edible coatings (ECs) containing piscicolin CM22 on raw tuna and salmon stored at 4 °C and 8 °C. Both gelatin-CM22 and chitosan-CM22 coatings demonstrated significant reductions in enterobacterial counts. For tuna stored at 4 °C, chitosan-CM22 reduced counts to approximately 1 log CFU/g on day 3, compared to the control at around 4 log CFU/g. Gelatin-CM22 also showed significant inhibition, reducing counts to about 1.5 log CFU/g. Similar results were observed for salmon, with chitosan-CM22 reducing counts below 1 log CFU/g, while controls reached nearly 5 log CFU/g. At 8 °C, both coatings continued to exhibit strong antimicrobial activity, maintaining low enterobacterial counts compared to significantly higher counts in control samples.

**Figure 3:**
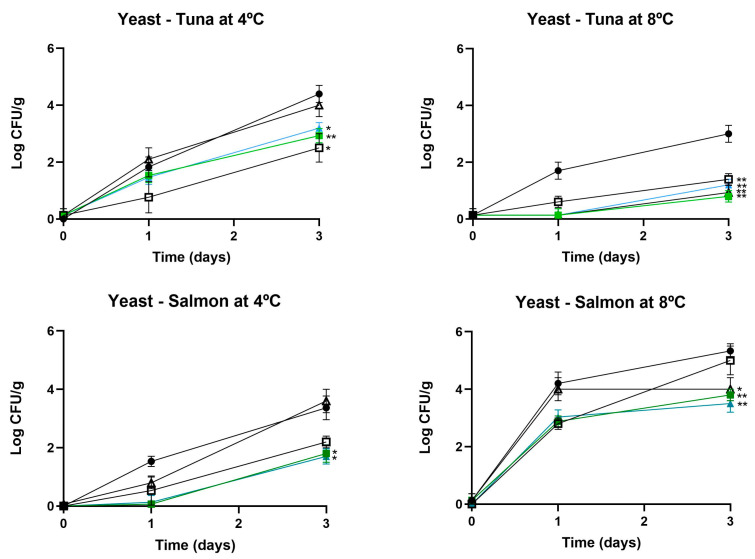

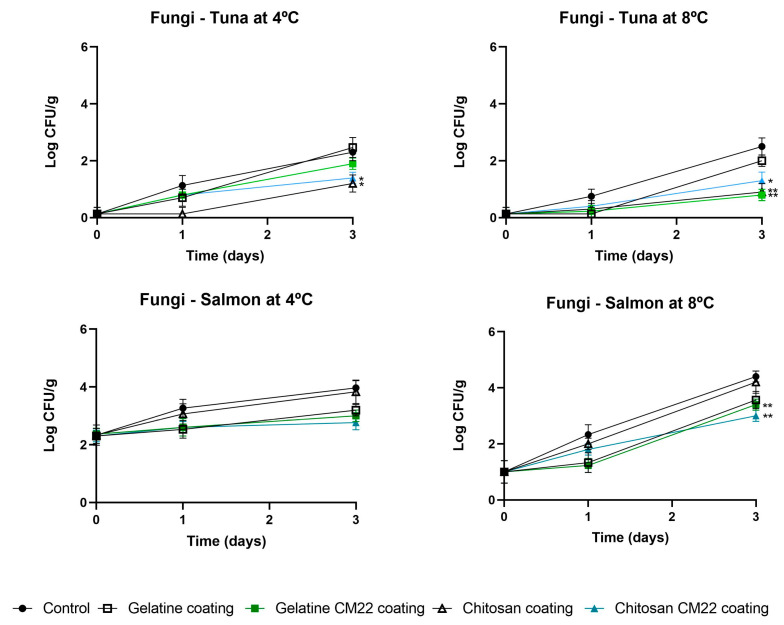
Effect of treatments with different edible coatings (ECs) containing piscicolin CM22 on yeast and fungi in raw tuna and salmon stored at 4 °C and 8 °C. Values are the average ± SD (error bars) from independent experiments. Significant differences compared to control: * *p* < 0.05; ** *p* < 0.01.

The results in Figure 3 highlight the effectiveness of ECs in controlling yeast and fungi in raw tuna and salmon stored at 4 °C and 8 °C, although this effect was less pronounced compared to bacteria. For yeast control, chitosan-CM22 was the most effective, reducing yeast counts to approximately 1 log CFU/g in tuna at 4 °C on day 3, compared to 3.5 log CFU/g in untreated samples (*p* < 0.01). Gelatin-CM22 also provided some inhibition, reducing counts to around 1.5 log CFU/g. At 8 °C, chitosan-CM22 kept yeast counts in tuna around 1 log CFU/g, while controls increased to approximately 4 log CFU/g. Regarding fungi, tuna stored at 4 °C and treated with chitosan-CM22 coatings showed significant reductions in fungal counts. Chitosan-CM22 maintained counts to about 1 log CFU/g by day 3, compared to the control at 2.5 log CFU/g. Finally, at 8 °C, tuna treated with chitosan-CM22 maintained fungal counts around 1 log CFU/g, while control samples increased to about 2.5 log CFU/g.

Since bacteriocins typically do not possess antifungal properties, the observed protective effect against fungi and yeasts cannot be attributed to these compounds. Instead, it is more likely attributable to the inherent antifungal properties of chitosan itself. Chitosan is well-documented for its broad-spectrum antifungal activity [32,33,34,35]. Additionally, the barrier effect provided by the ECs plays a crucial role, as it impedes the proliferation of spoilage microorganisms, particularly aerobic ones [36]. This dual action—direct antifungal activity of chitosan combined with the protective barrier effect—likely accounts for the observed reduction in fungal and yeast growth.

#### 3.2.2. Influence in Technological Properties

The pH stability for both salmon and tuna with different ECs at storage temperatures of 4 °C and 8 °C (Table 4) revealed that the variations in pH were minimal and statistically insignificant. For salmon, the treatments including gelatin coating (GC), gelatin coating with CM22 (GC-CM22), chitosan coating (CC), and chitosan coating with CM22 (CC-CM22) showed only minor deviations from the control group. At 4 °C, the pH remained largely consistent across all treatments, with fluctuations that did not substantially impact overall pH stability. Similarly, at 8 °C, while there were slight changes in pH values among the treatments, these differences were not significant. These results suggest that, under the tested conditions, the use of these ECs does not substantially alter the pH of the products.

**Figure 4:**
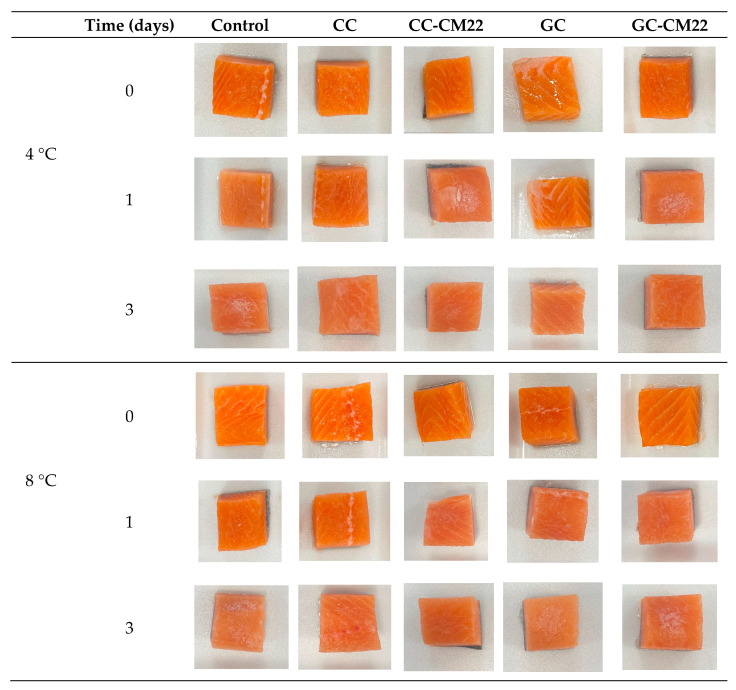
Photographs of salmon illustrating the progression of color under various treatments, stored at 4 °C and 8 °C.

Color is a critical attribute of fresh salmon and tuna, greatly affecting consumer perception and its marketability [37]. Maintaining the natural color during storage is essential, as any variations may indicate spoilage or decreased quality, making it a key parameter to monitor throughout the shelf life of these seafood products. The analysis of the color evolution in fresh salmon under different treatments revealed significant variations in the colorimetric parameters L*, a*, and b* (Table 5 and Figure 4). In terms of lightness (L*), the control exhibited a notable decrease in brightness, with values reduced to 42.41 and 38.66 on day 3 at 4 °C and 8 °C, respectively. In contrast, the chitosan coating (CC) and gelatin coating (GC) demonstrated better color preservation. The chitosan coating maintained relatively stable lightness at 4 °C (52.20 on day 3) and at 8 °C (48.65 on day 3) compared to the control. The gelatin coating also showed a positive effect, with L* values indicating less loss of brightness at 8 °C.

Regarding a* and b* parameters (red and yellow intensity), the control exhibited a significant decrease in redness over time, with a* values falling from 24.35 to 11.49 at 8 °C on day 3. In contrast, the chitosan and gelatin coatings demonstrated better preservation of red color, with the gelatin coating maintaining higher a* values at 4 °C and the chitosan coating showing relative stability at 8 °C. For the b* parameter, which measures yellow intensity, both the control and the treatments experienced a loss of yellow intensity; however, the chitosan and gelatin coatings were more effective in preserving this parameter.

Ultimately, it is important to highlight that the incorporation of piscicolin CM22 into the coatings did not substantially affect color preservation. The values for L*, a*, and b* were similar between coatings with and without CM22, demonstrating that this additive had a negligible impact on maintaining color stability.

**Figure 5:**
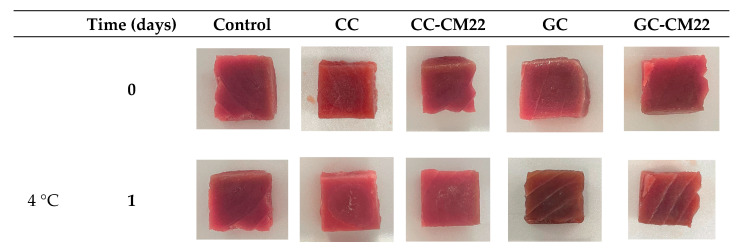

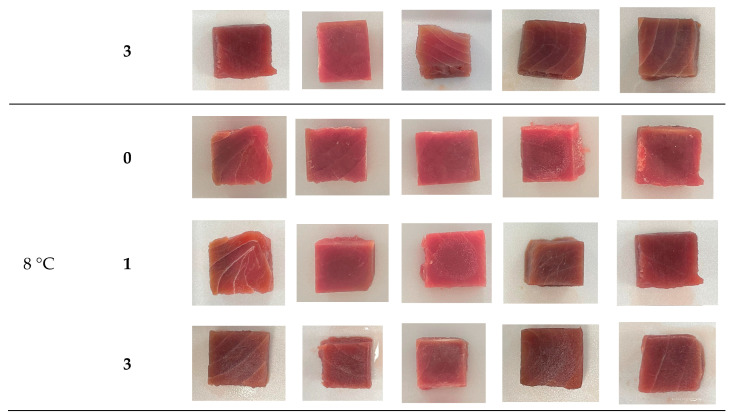
Photographs of tuna illustrating the progression of color under various treatments, stored at 4 °C and 8 °C.

The color values for tuna during shelf life at 4 °C and 8 °C, as detailed in Table 6 (Figure 5), reveal that chitosan coatings (CC and CC-CM22) were highly effective in preserving lightness (L*), particularly at 4 °C, where values remained between 38.16 and 40.04. In contrast, the control and gelatin coatings showed significant reductions in lightness, especially at 8 °C, where the control’s L* value fell to 18.48 by day 3, potentially impacting perceived freshness. Chitosan coatings also maintained a superior red color intensity (a*) compared to gelatin coatings. Although the preservation of yellow color intensity (b*) showed less variation among treatments, chitosan coatings slightly outperformed the control and gelatin coatings, indicating a modest benefit in maintaining yellow color.

The results obtained suggest that gelatin coatings (GC and GC-CM22) could be counterproductive in this case, accelerating color degradation faster than the control and potentially negatively affecting the perceived freshness and quality of the final product. The data imply that while both chitosan and gelatin coatings might be suitable for fresh salmon, the application of gelatin-based coatings for fresh tuna would be entirely inadvisable.

These findings underscore the critical importance of conducting tailored sensory studies for each specific food matrix, offering valuable insights into the seafood industry. Furthermore, they emphasize the necessity of developing preservation strategies that are not only effective but also align with the high-quality standards expected by consumers.

### 3.3. Challenge Test

**Figure 6:**
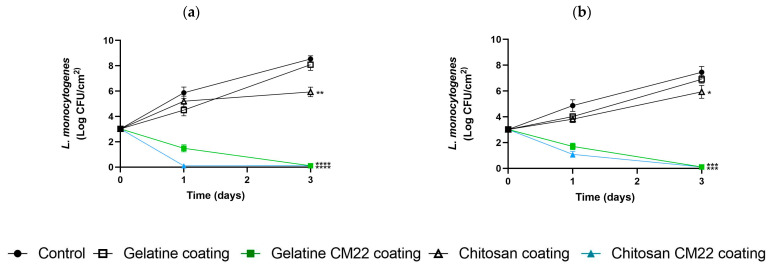
Effect of treatments with edible coatings (ECs) with and without piscicolin CM22 on the viability of *Listeria monocytogenes* in raw tuna (**a**) and raw salmon (**b**) stored at 4 °C and packaged in MAP. Values are the mean ± SD (error bars) from independent experiments. * *p* < 0.05; ** *p* < 0.01; *** *p* < 0.001; **** *p* < 0.0001 compared to control.

Challenge tests were conducted by superficially inoculating *L. monocytogenes* to assess the level of protection offered by each coating, both with and without piscicolin CM22, against the pathogen. Figure 6 shows the results expressed in Log CFU/cm^2^.

For tuna, the results indicated that ECs containing piscicolin CM22 were significantly more effective in reducing *L. monocytogenes* compared to ECs without bacteriocin and the control. The chitosan coating with piscicolin CM22 (CC-CM22) was the most effective, achieving a drastic and significant reduction in bacterial counts in three days. The gelatin coating with piscicolin CM22 (GC-CM22) also showed a notable and significant reduction, with bacterial counts decreasing below the limit of detection. However, the elimination was not complete in both cases, as the pre-enrichment test returned positive for *Listeria*. In contrast, the coatings without piscicolin CM22 (CC and GC) and the control exhibited a significant increase in bacterial counts, reaching nearly 9 log CFU/cm^2^ in the control.

For salmon, a similar pattern was observed. The coatings containing piscicolin CM22 (CC-CM22 and GC-CM22) demonstrated the highest effectiveness, significantly reducing *L. monocytogenes* counts below the limit of detection after three days of storage, compared to the coatings without piscicolin CM22 (CC and GC) and the control, which showed significant increases in bacterial counts (approx. 8 log CFU/cm^2^). Despite the significant reduction observed, the enrichment test again tested positive for the presence of *L. monocytogenes*.

**Figure 7:**
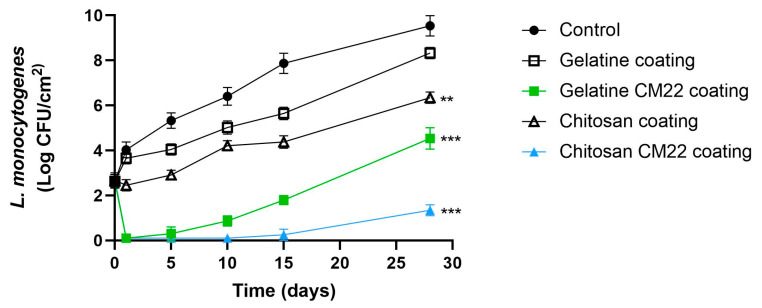
Effect of treatments with piscicolin CM22 on the viability of *Listeria monocytogenes* in vacuum-packed smoked salmon stored at 4 °C. Values are the average ± SD (error bars) of independent experiments. ** *p* < 0.01; *** *p* < 0.001; respect to control.

Finally, challenge tests were conducted on smoked salmon, which is characterized by a longer shelf life and different packaging and preservation conditions. The primary aim of these assays was to evaluate whether the efficacy of the coatings (ECs) remained effective over time. To this end, vacuum-packed smoked salmon was superficially inoculated with *L. monocytogenes*. Figure 7 presents the results expressed in Log CFU/cm^2^.

The control samples exhibited a significant increase in bacterial counts over the 30 days, reaching nearly 9 log CFU/cm^2^. Similarly, the samples treated with chitosan (CC) and gelatin (GC) coatings without piscicolin CM22 also showed important increases in *Listeria* counts, reaching approximately 6 and 8 log CFU/cm^2^, respectively. In contrast, the samples treated with chitosan and piscicolin CM22 (CC-CM22) and gelatin with piscicolin CM22 (GC-CM22) demonstrated a significant reduction in bacterial counts compared to the controls. However, it is noteworthy that *Listeria* began to recover after day 15 of storage at 4 °C (Figure 7), with concentrations reaching approximately between 1 and 4 log CFU/cm^2^. This recovery of the pathogen could be due to the repair of *Listeria* cells with sublethal injury [38] or to a loss of stability of piscicolin CM22 over an extended storage time. Therefore, further studies are needed to investigate this phenomenon.

## 4. Discussion

The use of ECs has rapidly expanded across nearly all sectors of the food industry, primarily due to their significant ability to enhance food safety and preservation. ECs function as physical barriers that protect the microbiological quality of food, thereby extending its shelf life [18]. This protective barrier is achieved through the film-forming substances used in their production, which not only safeguard the food but may also impart inherent antimicrobial properties, such as those found in chitosan [32,39]. Furthermore, ECs offer the distinct advantage of being able to incorporate antimicrobials, such as bacteriocins, which have been extensively studied as an effective biopreservation strategy for controlling pathogenic and spoilage bacteria [33]. In this study, we functionalized chitosan-based ECs with bacteriocins, which are widely used to extend shelf life due to their antimicrobial properties, with successful applications in meat and seafood products [18,40,41,42]. Similarly, fish gelatin ECs have demonstrated the ability to inhibit microbial growth, providing comparable benefits in different seafood [4].

Numerous researchers have incorporated bacteriocins into ECs. For example, nisin has been shown to be effective in whey protein-based coatings for controlling pathogens such as *Listeria* and spoilage microorganisms like *Bacillus thermosphacta* [24]. Furthermore, other studies have reported the anti-Listerial activity of an EC based on corn protein supplemented with nisin in turkey frankfurter [43]. Similar to our work, trials have been conducted with nisin in fish gelatin-based ECs applied to ham and bologna [44], as well as turkey-type sausages [45].

As previously mentioned, one of the significant advantages of ECs is their potential to serve as carriers of antimicrobials [24]. In this context, the combined effects of chitosan with other antimicrobial agents, such as organic acids, essential oils, or bacteriocins, have also been investigated. Beverlya et al. (2008) reported the anti-Listeria properties of chitosan-based coatings combined with acetic and lactic acids [46]. Similarly, Ojagh et al. (2010) found that applying a chitosan coating enriched with cinnamon oil reduced *L. monocytogenes* counts on refrigerated rainbow trout [47].

In our study, we have demonstrated the potential of incorporating the bacteriocin piscicolin CM22 into two types of coatings as a promising technology for food preservation. However, many aspects of their efficacy remain unknown. Although bacteriocins are effective at low concentrations, they often exhibit low in vivo stability and are susceptible to degradation by proteolytic enzymes [48]. Jack et al. (1996) observed that the bactericidal activity of piscicolin 126 was not destroyed by exposure to elevated temperatures at low pH values; however, this activity was lost at high pH values, especially when high pH was combined with elevated temperatures [49]. These findings align with our in vitro results, where piscicolin CM22 was completely degraded by proteolytic enzymes and exhibited instability under highly alkaline conditions and high temperatures.

In food trials, edible coatings enriched with piscicolin CM22 have demonstrated promising results in maintaining technological properties. Specifically, these coatings effectively preserve optimal pH and color even under suboptimal storage conditions. Quality degradation in fresh tuna and salmon is often associated with increased pH levels due to the formation of alkaline compounds such as trimethylamine, ammonia, and other basic volatile substances produced during spoilage [50,51]. Additionally, as noted earlier, color is a crucial attribute for both fresh salmon and tuna, significantly impacting consumer perception and marketability [37]. The ability of chitosan-based coatings to maintain the natural color of salmon and tuna during storage is a notable achievement. These results underscore the importance of assessing technological properties for each specific food product, as alterations to critical attributes such as color in raw tuna can significantly impact consumer acceptance.

Beyond the direct findings, our study also revealed that both edible coatings (ECs) substantially improved the microbiological quality of raw tuna and salmon during storage at 4 °C and 8 °C. Notably, significant reductions were observed in the counts of enterobacteria, fungi, and yeasts. This is particularly remarkable considering that most bacteriocins produced by LAB are predominantly effective against Gram-positive bacteria. The broader antimicrobial effect observed in our study may be attributed to the intrinsic antimicrobial properties of the coating materials themselves, as well as the barrier effect that restricts microbial growth. Chitosan, in particular, is well-documented for its broad-spectrum antimicrobial activity and its effectiveness as a preservative in film-forming edible coatings [52]. While the direct antimicrobial action of piscicolin CM22 was not evident, it is plausible that a synergistic interaction between chitosan and piscicolin CM22 contributed to the observed effects. Synergism has been previously reported in studies combining chitosan with enzymes like lysozyme [53] and bacteriocins such as nisin [54], where the combined application enhanced antimicrobial efficacy against both Gram-positive and Gram-negative bacteria.

The most notable results were obtained in the challenge tests against *L. monocytogenes*, which demonstrated a significant reduction of the pathogen in raw tuna, raw salmon, and smoked salmon. Similar findings were reported by Wan et al. (1997) who described the ability of piscicolin 126 in milk and Camembert cheese to inhibit *L. monocytogenes* [55]. Additionally, studies focused on the treatment of listeriosis, such as the work by Igham (2003), have shown in vivo protection provided by piscicolin against *Listeria* in infected mice [11]. It is also important to emphasize that piscicolins are not the only bioactive compounds of interest produced by *C. maltaromaticum* [56]; other peptides and bacteriocins from this species have also demonstrated significant activity against *Listeria* and other food spoilage microorganisms [57,58,59].

In summary, given the well-documented vulnerability of raw or minimally processed fish products such as raw tuna, salmon, and smoked salmon to pathogens as *L. monocytogenes* (particularly due to its ability to grow at refrigeration temperatures) [60,61,62], this study presents innovative developments using bacteriocins produced by psychrotolerant LAB strains as a preservation strategy for refrigerated fish products. Additionally, the efficacy of these ECs containing piscicolin CM22 at both 4 °C and 8 °C (where 8 °C indicates potential abuse during cold chain disruptions) demonstrates their robustness under various conditions. Furthermore, these ECs have been evaluated within the framework of multiple barrier technology, which integrates different preservation methods such as refrigeration, modified atmosphere packaging (MAP), and vacuum packaging [63]. Thus, the new ECs align well with this approach, providing an additional barrier that is compatible with current preservation technologies.

Overall, the integration of piscicolin CM22 into ECs represents a significant advancement in biopreservation strategies, leveraging the natural antimicrobial properties of bacteriocins to enhance the quality and safety of seafood products. Thus, future research should focus on optimizing these coatings for different food matrices and validating their efficacy under real-world conditions to fully realize their potential in food preservation.

## 5. Conclusions

This study underscores the substantial potential of incorporating piscicolin CM22, a novel bacteriocin derived from a psychrotolerant *C. maltaromaticum* strain, into chitosan-based and fish gelatin-based edible coatings (ECs) to enhance the quality and safety of fish products. The findings demonstrate that these ECs significantly improved microbiological quality and extended the shelf life of raw fish stored at both 4 °C and 8 °C. Moreover, chitosan-based ECs maintained pH stability and enhanced color retention, thereby preserving the sensory and technological attributes of the fish products. Conversely, fish gelatin-based coatings were found to alter the color of raw tuna. Additionally, both ECs exhibited potent anti-Listerial activity, significantly reducing the microbial counts of the pathogen in raw tuna, raw salmon, and smoked salmon. These results suggest that the integration of natural antimicrobial agents such as piscicolin CM22 into biopolymer-based coatings represents a promising biopreservation strategy, meeting the increasing consumer demand for safer and more natural food products.

## Figures and Tables

**Table 1:** Antimicrobial activity stability of piscicolin CM22 under different conditions of temperature, pH, and treatment with enzymes.

Treatments	Bacteriocin Activity ^a^
Control (only bacteriocin)	26 ± 0.5
**Heat**	
60 °C for 30 min	26 ± 1
80 °C for 30 min	25 ± 0.5
100 °C for 30 min	20 ± 1
121 °C for 15 min	-
**pH**	
2	25 ± 0.5
3	26 ± 1
5	26 ± 1
7	26 ± 0.5
9	35 ± 0.2
10	20 ± 0.5
11	-
**Enzymes**	
Trypsin, papain, and proteinase K	-

^a^ Diameter of inhibition zone in mm against *L. monocytogenes* DSM 112142.

**Table 2:** Physicochemical characteristics of Edible Coating (EC) with and without piscicolin CM22. Thickness of the Films (TFs), Films Solubility (FS), Films Water Swelling (FWS), Films Water Vapor permeability (WVP).

Edible Coatings (ECs)	TF μm	FS, %	FWS, %	WVP, mg cm^−2^ h^−1^
Gelatin coating (GC)	32.5 ± 0.6	8.7 ± 1.2	63.4 ± 2.6	8.7 ± 1.3
Gelatin-CM22 coating (GC-CM22)	31.9 ± 1.3	7.8 ± 0.9	60.9 ± 4.1	8.8 ± 1.7
Chitosan coating (CC)	21.7 ± 0.9	5.3 ± 0.7	39.7 ± 1.4	4.2 ± 1.1
Chitosan-CM22 coating (CC-CM22)	22.3 ± 1.8	4.8 ± 0.6	41.2 ± 2.3	4.3 ± 1.5

**Table 3:** In vitro effectiveness of different ECs against several *Listeria* strains, determined by the size of inhibition zones using the diffusion method in solid media. Results expressed as mm.

Strain	Inhibition of Halo (mm)				
	GC	GC-CM22	CC	CC-CM22	CM22
*L. monocytogenes* DZSM 112143	0	19 ± 1	10 ± 1	28 ± 1	24 ± 1
*L. monocytogenes* DMC 04	0	19 ± 1.5	11 ± 1	29 ± 1.4	25 ± 1.4
*L. monocytogenes* DMC 05	0	19 ± 2	11 ± 1.2	29 ± 2	22 ± 2
*L. monocytogenes* CECT 4032	0	22 ± 1	11 ± 1.2	27 ± 1	25 ± 1
*L. monocytogenes* CECT 5342	0	23 ± 1.3	12 ± 1	26 ± 1.5	26 ± 1.5

**Table 4:** Effect of treatments of edible coatings (ECs) with piscicolin CM22 on pH of raw tuna and salmon stored at 4 °C and 8 °C.

		Time (Days)		
Salmon		0	1	3
Control		6.72	6.72	6.85
Gelatin coating (GC)		6.72	6.72	6.83
Gelatin coating with CM22 (GC-CM22)	4 °C	6.94	6.94	6.75
Chitosan coating (CC)		6.79	6.79	6.77
Chitosan coating with CM22 (CC-CM22)		6.87	6.87	6.86
Control		6.85	6.85	7.15
Gelatin coating (GC)		6.88	6.88	6.81
Gelatin coating with CM22 (GC-CM22)	8 °C	6.87	6.87	6.81
Chitosan coating (CC)		6.75	6.75	6.78
Chitosan coating CM22 (CC-CM22)		6.73	6.73	6.79
**Tuna**				
Control		6.54	6.54	6.59
Gelatin coating (GC)		6.38	6.38	6.67
Gelatin coating with CM22 (GC-CM22)	4 °C	6.71	6.71	6.72
Chitosan coating (CC)		6.38	6.38	6.35
Chitosan coating with CM22 (CC-CM22)		6.42	6.42	6.64
Control		6.57	6.57	6.56
Gelatin coating (GC)		6.53	6.53	6.77
Gelatin coating CM22 (GC-CM22)	8 °C	6.68	6.68	6.76
Chitosan coating (CC)		6.74	6.74	6.79
Chitosan coating with CM22 (CC-CM22)		6.49	6.49	6.77

**Table 5:** Color modification of treatments with edible coatings (ECs) over time in raw salmon stored at 4 and 8 °C.

Parameter		Time (Days)	Control	CC	CC-CM22	GC	GC-CM22
L*		0	50.77 ± 0.87	52.94 ± 0.87	52.93 ± 0.87	51.29 ± 0.87	51.31 ± 0.87
	4 °C	1	53.68 ± 1.92	51.68 ± 2.34	52.57 ± 0.98	51.15 ± 3.01	51.4 ± 3.01
		3	42.41 ^a^ ± 2.03	52.20 ^b,c^ ± 3.73	55.66 ± 4.03	50.35 ^c^ ± 2.27	50.38 ^b^ ± 2.27
		0	52.63 ± 1.08	52.94 ± 1.37	52.94 ± 0.13	51.25 ± 0.88	51.11 ± 0.88
	8 °C	1	42.49 ^a^ ± 0.97	43.83 ^a^ ± 1.02	49.33 ^b^ ± 0.90	50.35 ^b^ ± 0.53	50.96 ^b^ ± 0.53
		3	38.66 ^a^ ± 0.85	48.65 ^b^ ± 0.48	48.55 ^b^ ± 1.47	50.55 ^c^ ± 0.48	49.81 ^b,c^ ± 0.48
a*		0	24.35 ^b^ ± 0.98	21.81 ^a^ ± 0.98	21.81 ^a^ ± 0.98	24.76 ^b^ ± 0.98	24.93 ^b^ ± 0.98
	4 °C	1	11.15 ^a^ ± 0.75	15.65 ^b^ ± 0.10	15.85 ^b^ ± 1.31	19.85 ^c^ ± 0.7	19.82 ^c^ ± 0.7
		3	12.71 ^a^ ± 0.65	14.11 ^a,b^ ± 0.85	13.50 ^a^ ± 1.44	17.69 ^b^ ± 1.13	17.57 ^b^ ± 1.13
		0	23.44 ± 1.75	21.81 ± 1.01	21.81 ± 0.66	24.27 ± 0.43	24.96 ± 0.43
	8 °C	1	13.42 ^a^ ± 0.41	18.55 ^b^ ± 0.74	17.58 ^b^ ± 0.34	18.21 ^b^ ± 0.65	13.20 ^a^ ± 0.65
		3	11.49 ^a^ ± 0.13	15 ^b^ ± 0.34	15.52 ^b^ ± 0.42	11.51 ^a^ ± 0.70	11.52 ^a^ ± 0.70
b*		0	13.35 ± 0.40	14.46 ± 0.40	14.46 ± 0.40	14.9 ± 0.40	14.58 ± 0.40
	4 °C	1	12.58 ± 0.68	14.71 ± 0.67	13.75 ± 1.34	14.88 ± 1.75	13.71 ± 1.75
		3	6.31 ^a^ ± 0.88	15.27 ^b^ ± 1.88	13.3 ^b^ ± 1.11	13.63 ^b^ ± 0.58	13.64 ^b^ ± 0.58
		0	13.52 ± 0.33	13.46 ± 0.69	13.46 ± 0.47	14.81 ± 0.23	13.57 ± 0.23
	8 °C	1	8.79 ^a^ ± 0.24	11.97 ^b^ ± 0.27	13.28 ^b^ ± 0.17	12.68 ^b^ ± 0.47	13.68 ^b^ ± 0.47
		3	1.49 ^a^ ± 0.1	9.51 ^b^ ± 0.3	9.48 ^b^ ± 0.26	10.48 ^b^ ± 0.46	10.48 ^b^ ± 0.46

Different letters indicate statistically significant differences (*p* < 0.05) between treatments.

**Table 6:** Color modification of treatments with edible coatings (ECs) over time in raw tuna stored at 4 and 8 °C.

Parameter		Time (Days)	Control	CC	CC-CM22	GC	GC-CM22
L*		0	27.05 ^a^ ± 0.87	40.55 ^b^ ± 0.87	40.55 ^b^ ± 0.87	27.76 ^a^ ± 0.87	27.45 ^a^ ± 0.87
	4 °C	1	26.62 ^a^ ± 1.92	38.16 ^b^ ± 2.34	38.57 ^b^ ± 0.98	28.79 ^a^ ± 3.01	28.44 ^a^ ± 3.01
		3	25.17 ^a^ ± 2.03	38.60 ^b^ ± 3.73	40.04 ^b^ ± 4.03	28.6 ^a^ ± 2.27	28.31 ^a^ ± 2.27
		0	27.5 ^a^ ± 1.08	42.55 ^b^ ± 1.37	42.55 ^b^ ± 0.13	27.5 ^b^ ± 0.88	27.48 ^a^ ± 0.88
	8 °C	1	11.50 ^a^ ± 0.97	37.90 ^c^ ± 1.02	33.66 ^c^ ± 0.90	22.2 ^b^ ± 0.53	23.66 ^b^ ± 0.53
		3	18.48 ^a^ ± 0.85	38.45 ^b^ ± 0.48	38.47 ^b^ ± 1.47	18.46 ^a^ ± 0.48	18.49 ^a^ ± 0.48
a*		0	16.24 ^b^ ± 0.98	15.47 ^b^ ± 0.98	15.47 ^b^ ± 0.98	5.71 ^a^ ± 0.98	5.13 ^a^ ± 0.98
	4 °C	1	16.89 ^b^ ± 0.75	16.07 ^b^ ± 0.10	16.35 ^b^ ± 1.31	9.61 ^a^ ± 0.7	9.97 ^a^ ± 0.7
		3	15.14 ^b^ ± 0.65	16.51 ^b^ ± 0.85	16.24 ^b^ ± 1.44	11.52 ^a^ ± 1.13	12.4 ^a^ ± 1.13
		0	16.64 ^b^ ± 1.75	16.79 ^b^ ± 1.01	16.79 ^b^ ± 0.66	5.99 ^a^ ± 0.43	5.08 ^a^ ± 0.43
	8 °C	1	9.24 ^a^ ± 0.41	14.49 ^b^ ± 0.74	14.63 ^b^ ± 0.34	8.11 ^a^ ± 0.65	8.49 ^a^ ± 0.65
		3	0.52 ^a^ ± 0.13	12.52 ^b^ ± 0.34	12.52 ^b^ ± 0.42	0.53 ^a^ ± 0.70	0.53 ^a^ ± 0.70
b*		0	5.29 ^a^ ±0.40	5.47 ^b^ ± 0.40	5.55 ^b^ ± 0.40	2.16 ^a^ ± 0.40	2.81 ^a^ ± 0.40
	4 °C	1	6.66 ^a^ ±0.68	5.06 ^a^ ± 0.67	6.04 ^a^ ± 1.34	6.60 ^a^ ± 1.75	10.27 ^b^ ± 1.75
		3	2.07 ^a^ ±0.88	4.51 ^b^ ± 1.88	4.44 ^b^ ± 1.11	1.51 ^a^ ± 0.58	15.68 ^c^ ± 0.58
		0	5.78 ^a^ ±0.33	5.14 ^b^ ± 0.69	5.14 ^b^ ± 0.47	2.05 ^a^ ± 0.23	2.77 ^a^ ± 0.23
	8 °C	1	6.94 ^a^ ±0.24	5.14 ^b^ ± 0.27	5.11 ^b^ ± 0.17	1.17 ^a^ ± 0.47	1.43 ^a^ ± 0.47
		3	1.48 ^a^ ±0.1	4.51 ^b^ ± 0.3	4.48 ^b^ ± 0.26	1.52 ^a^ ± 0.46	1.49 ^a^ ± 0.46

Different letters indicate statistically significant differences (*p* < 0.05) between treatments.

## Data Availability

The original contributions presented in the study are included in the article, further inquiries can be directed to the corresponding authors.
